# Analysis of pig trading networks and practices in Uganda

**DOI:** 10.1007/s11250-018-1668-6

**Published:** 2018-08-02

**Authors:** C. Atherstone, R. G. Galiwango, D. Grace, S. Alonso, N. K. Dhand, M. P. Ward, S. M. Mor

**Affiliations:** 1 Sydney School of Veterinary Science, University of Sydney, Sydney, NSW 2006 Australia; 2 International Livestock Research Institute, PO Box 24384, Kampala, Uganda; 3 Uganda National Farmers Federation, PO Box 6213, Kampala, Uganda; 4 International Livestock Research Institute, PO Box 30709, Nairobi, Kenya; 5 International Livestock Research Institute, PO Box 5689, Addis Ababa, Ethiopia

**Keywords:** Pig, Uganda, Trading, Business practices, Health reporting

## Abstract

East Africa is undergoing rapid expansion of pig rearing, driven by increasing pork consumption. Introduction and expansion of pig production systems in this biodiverse landscape may create new risks, including zoonotic pathogen transmission. Historically, biosecurity measures have primarily been focused at farm level, ignoring the important function pig traders fulfill between farmers and consumers. This study interviewed pig traders operating at Uganda’s only registered pork abattoir to describe their characteristics, business practices, biosecurity practices, and pig health management and reporting practices. All the traders were male, and nearly all (90.5%) relied on pig trading as their primary source of income. Most of the pigs brought for processing at the slaughterhouse were purchased from smallholder farms (87.3%). In addition, there was a significant difference in the high price paid per kilogram at farm gate by region (*P* = 0.005). High prices paid at farm gate were associated with holiday periods (*P* < 0.001), harvest season (*P* < 0.001), and drought (*P* < 0.001). Traders preferred buying live pigs from male farmers (88.9%) because they were considered the final decision makers and owned the pigs being sold. All pig traders were aware of clinical signs indicating a pig was sick. This study has provided baseline information on pig trader practices in Uganda. Improvements in local pork slaughterhouses and markets will benefit not only pig traders in accessing consistent customers but also individual pig farmers by increasing their market access. Finally, given their role as a link between farmers and consumers, traders would benefit from targeted inclusion in disease control and prevention strategies.

**Electronic supplementary material:**

The online version of this article (10.1007/s11250-018-1668-6) contains supplementary material, which is available to authorized users.

## Introduction

The domestic pig population in Uganda, currently estimated at 3.2 million, plays an essential economic and social role in a country, in which 70% of households derive some or all of their livelihoods directly from livestock (Uganda Bureau of Statistics 2008). Pig keeping has grown in popularity as a livelihood activity due to their high reproduction rates, rapid weight gain, potential to provide quick financial returns, and rising demand for pork. In the past 50 years, pork consumption has increased more than 20-fold, from an estimated annual per capita consumption of 0.14 kg in 1962 to 3.37 kg in 2013. Pork currently accounts for more than a third of the annual per capita meat consumption (Food and Agriculture Organization (FAO) 2011). Further, total pork consumption is projected to increase by 184% between 2000 and 2030 in Uganda due to human population growth (Food and Agriculture Organization 2011). The increase in pork consumption, while exceptionally rapid in Uganda, is not unique in the region. The Democratic Republic of Congo’s total consumption of pork is expected to increase by 100%, Tanzania by 32%, and Kenya by 25% between 2000 and 2030 (Food and Agriculture Organization 2011). Introduction and expansion of pig production systems in these biodiverse landscapes may create new risks, including pathogen transfer from pigs to humans (Ocaido et al. 2013; Wilson 2017; Food and Agriculture Organization (FAO) 2012; Atherstone et al. 2015; Hamill et al. 2013; Food and Agriculture Organization 2017). Of particular public health interest is the role of pigs in the zoonotic transmission of emerging pathogens to people (Atherstone et al. 2015; Vergara-Alert et al. 2017; Middleton and Westbury 2002; Ma et al. 2008; Kobinger et al. 2011; Conlan et al. 2012; McCormack and Allworth 2002; AbuBakar et al. 2004; Marsh et al. 2011). As pig traders form an important link between pig farms and pork customers, research informing their knowledge, attitudes, and practices is essential.

African swine fever (ASF) is considered the major infectious disease constraint to pig production in Africa (Penrith et al. 2013), and as such, research to date has focused on this infection. Penrith and Vosloo reported that outbreaks of ASF in new areas of Africa have almost all been associated with movement of domestic pigs and pig products (Penrith and Vosloo 2009). In Uganda, several studies to characterize practices associated with the occurrence and spread of ASF identified the collection of pigs and pig products from farms (Kabuuka et al. 2014), distribution of infected pork by traders (Dione et al. 2015), pig movements due to restocking and trade (Kalenzi Atuhaire et al. 2013; Nantima et al. 2015), free range movement of pigs on farms (Nantima et al. 2015) and trade of live pigs and pig products (Tejler and Teijler 2012) as risk factors. These studies focused on pig farmers and their perception of trading and pig movements. A limited number of studies have targeted pig traders, but again, these were restricted to knowledge and practices related to ASF transmission and control (Dione et al. 2016; Chenais et al. 2015; Muhangi et al. 2014). Despite the link between disease spread and pig movement, little is known about broader trading practices, motivations for buying, and patterns of purchases and sales of pigs in Uganda. Given pig traders’ important role in supplying pork for a rapidly expanding consumer base and linking farmers with consistent markets, a better understanding of their practices and motivations around purchasing, transportation, and pig health management is needed. This would assist in developing policies that specifically support traders and the important functions they serve while identifying suitable interventions to ensure a safe, reliable pork supply.

Therefore, the objectives of this study were to (1) describe pig trader characteristics, trading practices, biosecurity practices, pig health management, and reporting practices and (2) map source locations of pigs purchased to supply pork through the major abattoir in Uganda.

## Materials and methods

### Study area

Wambizzi Cooperative Society Limited is located in Nalukolongo, southwestern Kampala, Uganda’s capital city. Wambizzi was selected as it is the only registered pig abattoir in Uganda and has many pig traders supplying live pigs to meet the urban demand for pork. As a registered slaughterhouse, carcasses processed at Wambizzi are visually inspected by Kampala City Council Authority (KCAA) meat inspectors and stamped “fit for human consumption.” Pork processed at the abattoir is sold in the greater Kampala area to pubs, pork joints, hotels, butchers, supermarkets, and private organizations (non-governmental organizations, missions, and private individuals). The slaughterhouse has a capacity of 200 pigs/day, but the supply of pigs fluctuates substantially throughout the year (Roesel et al. 2016). According to slaughterhouse records, an average of 60,078 kg of pork was processed monthly from April 2014 to May 2015. Using the estimated annual per capita consumption of 3.37 kg of pork (Food and Agriculture Organization (FAO) 2011) and the 2014 Kampala population of 1.5 million (Uganda Bureau of Statistics 2016), Wambizzi produced roughly 14.3% of the pork consumed in Kampala during this time frame. Pig slaughtering also occurs in backyards and slaughter slabs to supply informal, roadside pork joints and butcheries.

Pig traders privately operating from Wambizzi use the abattoir facility to slaughter their pigs and have their pork inspected and stamped to meet requirements in the formal marketplace. Traders buy live pigs from farms/markets and aggregate them into groups for transport to the abattoir. During this interim period (farm gate to abattoir), pigs are under the ownership and care of traders. Once processed, traders sell the pork to their own customers in the sales building adjacent to the evisceration and inspection building. While membership with the abattoir is not required, traders pay a fee per pig (6000 UGX, i.e., 1.67 USD in 2017) to use the abattoir facilities (Roesel et al. 2016).

### Selection of traders

Pig traders were interviewed between October 2015 and October 2016, during periods corresponding with national holidays when the sale and consumption of pork increases (Roesel et al. 2016; Ouma et al. 2015). There is no formal register of pig traders operating at Wambizzi. In preparation for this research, a member of the research team with prior experience working with pig traders informally questioned traders on site over several days to develop a more recent estimate of the number of traders operating from Wambizzi. Based on this, the total number of traders operating at Wambizzi was estimated at 60. Thus, we aimed to interview 60 traders over the course of the study. A variety of methods were used to recruit participants, including direct approach and active-snowballing. Traders arriving at the abattoir were approached by a local member of the research team and initially asked about their interest in learning about the study. If the trader expressed interest, information on the scope and purpose of the research was provided orally. Traders who indicated that they were willing to participate in the research study were asked to give written consent to be interviewed. If the trader was unable to give written consent due to physical impairment or illiteracy, their thumbprint was provided in place of signature. Additional traders were identified by asking participants who had completed the interview for the name and contact information of other pig traders operating from Wambizzi. Furthermore, we observed trader brands on pigs at slaughter (e.g., number or letter carved on the animal at the time of purchase) and asked participants who had completed the interview if they could identify the trader who supplied the pig. The enumerator then contacted these newly identified pig traders to invite their participation in the research study.

### Data collection

A local enumerator with previous experience working with pig traders in Uganda was recruited and trained for data collection. A structured questionnaire was adapted from previous research conducted with pig traders by the International Livestock Research Institute (ILRI) in Uganda under the Smallholder Pig Value Chains Development Project (CGIAR Livestock and Fish Research Program 2015). The questionnaire captured information on pig trader characteristics, live pig buying practices, transportation practices, and pig health management. Traders also reported the sub-counties where they had purchased live pigs over the 12 months prior to the interview date. The questionnaire was developed in English and translated into the local language (Luganda). The questionnaire comprised primarily closed-ended questions to keep the interview to a maximum of 45 min. Open-ended questions regarding buying practices and clinical signs observed in pigs were included, with answers recorded exactly as the interviewee stated. The full questionnaire is included in the Supplementary Materials. The slaughter process started at 4 am each morning and preceded the selling of pork from 8 am to 10 am. The enumerator was on site by 6 am each morning to identify pig traders previously not interviewed. However, to ensure that the interview did not conflict with pig trader’s business, most interviews took place between 10 am and noon each day.

### Data analysis

Data from the questionnaires was entered into Epi Info 7.1 (Centers for Disease Control, Atlanta, GA, USA). Following data cleaning, data was exported to SPSS 24.0 (IBM Corp., Armonk, NY, USA) for analysis. Standard descriptive analysis was performed for categorical and quantitative variables describing pig trader characteristics, live pig purchasing practices, transportation practices, and pig health management. Population pyramid style graphs were prepared to compare pork demand and pork farm gate prices by months. Source locations (reported to the sub-county level) were entered into Microsoft Excel and checked for spelling accuracy. The sub-counties were then joined to the centroid of each sub-county polygon in the 2014 Global Administrative Unit Layers for Uganda (Food and Agriculture Organization, Rome, Italy) using ArcGIS 10.2 (Environmental Systems Research Institute, Redlands, CA, USA). The number of pig traders operating in each sub-county was mapped using graduated symbols.

Two binary outcome variables of interest were explored further, namely high price paid at farm gate over the last year (1/0) and low price paid at farm gate over the last year (1/0). Reasons given for prices paid were recoded into binary variables (yes/no) and used as explanatory variables. Univariable binomial logistic regression analyses were conducted to evaluate the associations of the binary explanatory variables with both the outcome variables. Explanatory variables with a *P* value < 0.15 were included in two multivariate regression models for high or low price paid to evaluate associations after adjusting for other variables in the model.

In addition, non-parametric analyses were conducted for four quantitative variables (high price paid per kilogram, low price paid per kilogram, number of live pigs bought during high demand weeks, and number of live pigs bought during low demand weeks) to identify significant differences by operating region (Kruskal-Wallis test), number of districts (Mann-Whitney *U* test), and number of regions (Mann-Whitney *U* test) traders purchased live pigs from. Operating region was identified based on the location pig traders reported purchasing live pigs in. Because price and number of pigs purchased were not identified by individual districts and many pig traders operated in multiple regions, operating region was binned into three categories: central only, eastern only, and all other regions (including western, northern, and responses that covered multiple regions). Number of regions and number of districts a pig trader operated in were recoded into two responses: above median and below median. Independent variables with significant differences (*P* < 0.05) were subject to post-hoc pairwise comparisons to identify which specific responses(s) were significantly different from each other.

## Results

A total of 63 interviews were conducted with pig traders operating from Wambizzi between October 2015 and October 2016. No traders declined participation in the study.

### Pig trader characteristics

Pig trader characteristics are shown in Table 1. All pig traders interviewed were male and ranged in age from 28 to 60 years (median 38 years; first quartile (Q1) = 34, third quartile (Q3) = 47). The median number of years working as a pig trader was 12 years (range 3 months–36 years; Q1 = 8, Q3 = 18.75). A large proportion (41.3%; 26/63) of participants had not completed primary school. Most pig traders were engaged in trading as their primary source of income (90.5%; 57/63) and described their business operation as fixed (96.8%; 61/63), meaning that they had established locations for buying live pigs and selling pork. Proximity to pork customers was the primary reason for having a fixed business operation (79.4%; 50/63) with most traders supplying pigs solely to Wambizzi (93.7%; 59/63). When asked about other pig traders operating in their areas, almost all the traders had competition for live pigs in their buying areas (98.4%; 62/63). Almost two thirds of the pig traders were not members of a trading group or cooperative (63.5%; 40/63).

**Table 1:** Characteristics of 63 pig traders interviewed at Wambizzi Cooperative Society slaughterhouse, Kampala, Uganda, 2015–2016

	Number (*n*)	Percentage (%)
Gender		
Male	63	100
Age		
20–29	2	3.2
30–39	31	49.2
40–49	21	33.3
≥ 50	5	7.9
Missing	4	6.3
Education		
School not attended	1	1.6
Primary school not completed	25	39.7
Primary school completed	17	27.0
Secondary school completed	14	22.2
University completed	3	4.8
Missing	3	4.8
Number of years working as pig trader		
0–9	20	31.7
10–19	28	44.4
20–29	9	14.3
≥ 30	3	4.8
Missing	3	4.8
Reason/s for selling pigs^a^		
Primary income	57	90.5
Secondary income	5	7.9
Missing	1	1.6
Position in business		
Owner	54	85.7
Employee	8	12.7
Business partner	1	1.6
Type of business		
Fixed	61	96.8
Mobile	2	3.2
Reasons for business type		
Close to pork customers (demand)	50	79.4
Close to pig farms (supply)	9	14.3
No competition	1	1.6
Missing	3	4.8
Group/cooperative membership		
No	40	63.5
Yes	23	36.5
Other traders in business area		
Yes	62	98.4
No	1	1.6
Location of pork sales		
Wambizzi	59	93.7
Other	3	4.8
Missing	1	1.6

^a^Multiple options could be selected, however no trader selected multiple options

### Live pig purchasing practices

Buying and transportation practices are shown in Table 2. Most traders sourced their pigs directly from smallholder farms (87.3%; 55/63). Only one trader reported purchasing pigs at a livestock market. When pig traders were asked about whom they prefer to purchase pigs from at the farm, 88.9% (56/63) preferred buying from men rather than from women. Reasons offered by traders who preferred to purchase from men included that men were the following: the decision makers on the farm, faster decision makers, and owned the pigs being sold. Further analysis to understand this gender preference was not possible because the number of responses for women were all less than 5.

**Table 2:** Buying and transportation practices of 63 pig traders interviewed at Wambizzi Cooperative Society slaughterhouse, Kampala, Uganda, 2015–2016

	Number (*n*)	Percentage (%)
Buying practices		
Farm type		
Smallholder farm	55	87.3
Own farm	3	4.8
Other^a^	2	3.2
Missing	3	4.8
Whom do you prefer buying pigs from?		
Men	56	88.9
Women	7	11.1
Time of day to buy pigs		
Afternoon	48	76.2
Morning	10	15.9
Midday	5	7.9
Transportation		
Vehicle type		
Lorry	38	60.3
Truck	25	39.7
Vehicle ownership		
Rented	57	90.5
Own	6	9.5
Frequency vehicle cleaned		
After each use	60	95.2
Daily	1	1.6
Missing	2	3.2
Cleaning products used^b^		
Water	63	100
Omo (laundry washing powder)	62	98.4
Method of animal waste disposal		
Heap at Wambizzi for farmers to collect	34	54.0
Throw away	17	27.0
Burn	4	6.3
Bury	1	1.6
Missing	1	1.6

^a^Livestock market (*n* = 1), commercial farm (*n* = 1)

^b^Multiple options could be selected

Lorries (trucks) were the most common type of vehicle used to transport pigs to the abattoir (60.3%; 38/63). The majority of traders rented the vehicles they used to transport pigs (90.5%; 57/63). Vehicles were cleaned after each use (95.2%; 60/63) using both water and laundry washing powder (98.4%; 62/63). None of the pig traders reported using bleach or any other type of disinfectant to clean their vehicles. The pig waste (feces, urine, bedding) left in the vehicle after transporting the pigs was most commonly heaped at Wambizzi for crop farmers to collect and use for compost in their gardens (54%; 34/63).

### Live pig volume and pricing

**Fig. 1:**
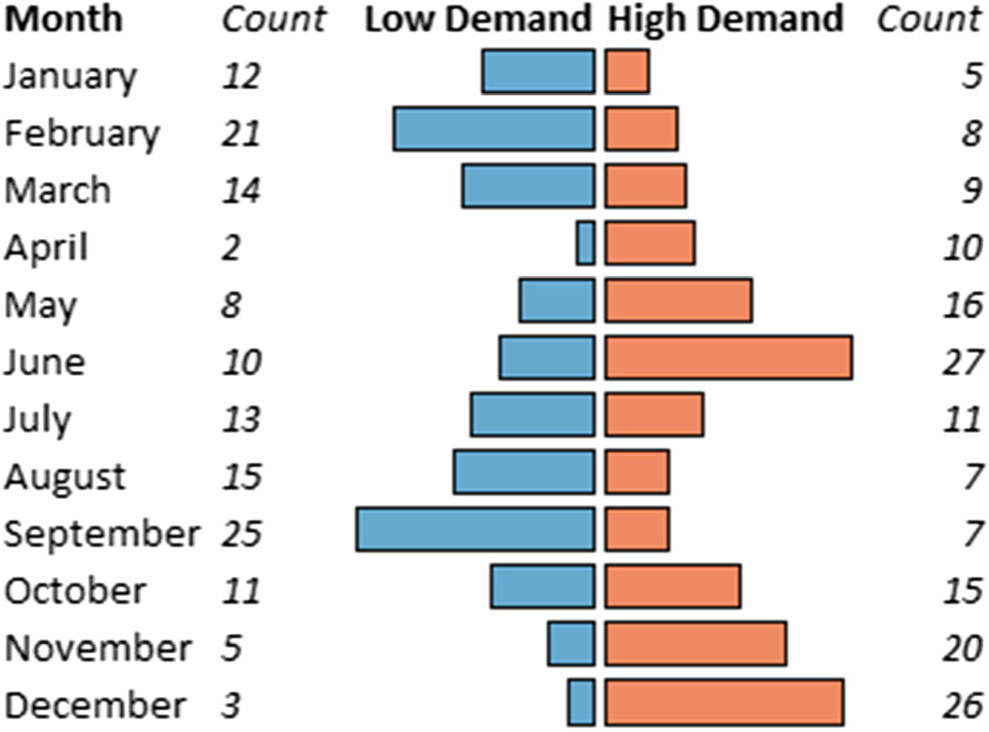
Months identified as having high and low customer demands for pork, as reported by 63 pig traders interviewed at Wambizzi Cooperative Society slaughterhouse, Kampala, Uganda, 2015–2016. Counts indicate the number of traders that selected that month as being associated with high or low demand, respectively

June and December were frequently identified as months with high customer demand for pork, whereas February and September were associated with low customer demand (Fig. 1). During months when demand for pork was low and high, respectively, traders bought a median of 28.5 pigs (range 3–140 pigs/week; Q1 = 20; Q3 = 45) and 77.5 pigs (range 5–260 pigs/week; Q1 = 50; Q3 = 120) per week.

**Fig. 2:**
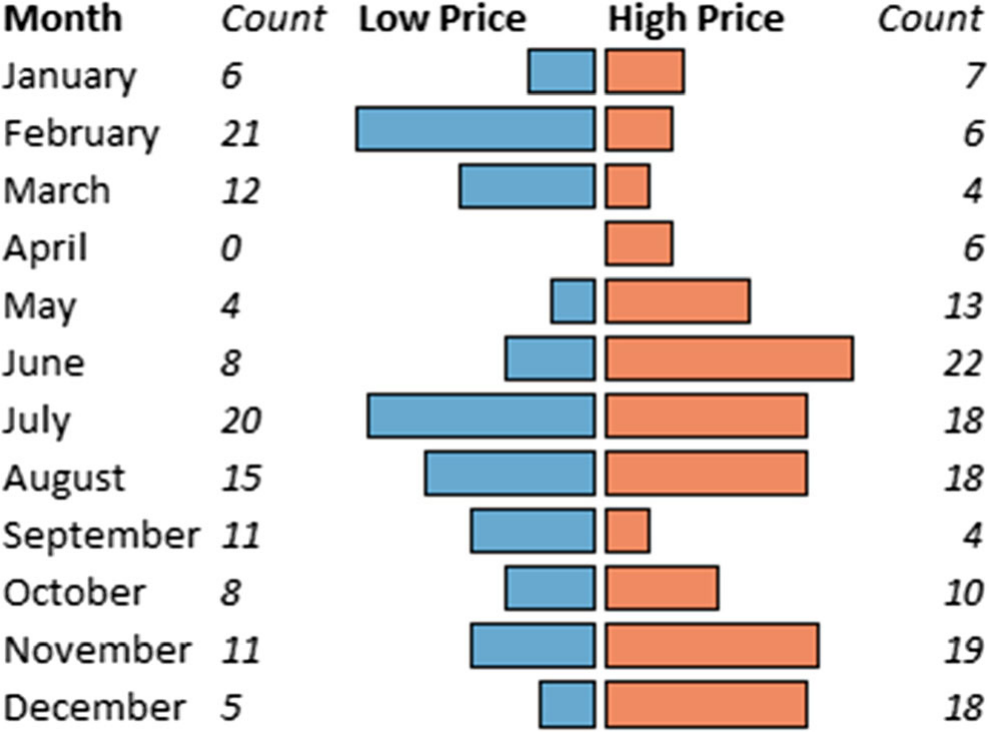
Months associated with high and low farm gate prices by 63 pig traders interviewed at Wambizzi Cooperative Society, Kampala, Uganda, 2015–2016. Counts indicate the number of traders that selected that month as being associated with high or low price, respectively

Figure 2 shows the months traders associated with paying high or low farm gate prices to purchase pigs. The median reported high prices at farm gate was 6000 UGX/kg (range 6000−8000 UGX/kg; 3590 UGX = 1 USD as of January 2017). When low prices were paid at farm gate, the median was 5000 UGX/kg (range 5000–7500 UGX/kg). Reasons for paying high and low prices at farm gate are shown in Table 3. In multivariate logistic regression, holiday period, crop/coffee harvesting season, and drought were significantly associated with high price paid, whereas drought, school fees due time, and sick pigs were significantly associated with low price paid.

**Table 3:** Reasons for high/low price paid by 63 pig traders interviewed at Wambizzi Cooperative Society slaughterhouse, Kampala, Uganda, 2015–2016. Explanatory variables with *P* < 0.15 in the univariable analyses were included in the final multivariable logistic regression model

Explanatory variable: reason for paying price	Outcome variable: high price paid					Outcome variable: low price paid				
	Frequency (%)	Unadjusted odds ratio (95% CI)	*P* value	Adjusted odds ratio (95% CI)	*P* value	Frequency (%)	Unadjusted odds ratio (95% CI)	*P* value	Adjusted odds ratio (95% CI)	*P* value
Holidays	27 (30.3)	4.59 (2.60–8.08)	< 0.001	8.70 (4.57–16.58)	< 0.001	4 (4.5)	^a^	^a^		
Crop/coffee harvesting season	19 (21.3)	2.29 (1.26–4.15)	0.01	5.01 (2.55–9.84)	< 0.001	2 (2.2)	^a^	^a^		
Drought	18 (20.2)	2.09 (1.14–3.81)	0.02	4.64 (2.35–9.18)	<0.001	23 (25.8)	3.27 (1.84–5.81)	< 0.001	13.36 (6.27–28.44)	< 0.001
School fees due	14 (15.7)	1.39 (0.73–2.65)	0.31	N/A	N/A	19 (21.3)	2.29 (1.26–4.15)	< 0.001	10.03 (4.63–21.73)	< 0.001
Sick pigs	8 (9.0)	0.65 (0.30–1.41)	0.27	N/A	N/A	34 (38.2)	8.23 (4.65–14.55)	< 0.001	27.24 (12.94–57.32)	< 0.001
Neighbor’s pigs are sick	2 (2.2)	^a^	^a^			5 (5.6)	0.37 (0.14–0.94)	0.04	^b^	^b^
Too many traders	1 (1.1)	^a^	^a^			0	N/A	N/A	N/A	N/A
Too many pigs for sale	0	N/A	N/A	N/A	N/A	2 (2.2)	^a^	^a^		

^*N/A* not applicable^

^a^Outcome variables with less than five responses were not including in the univariable analysis

^b^*P* value = 0.089. Explanatory variable removed from multivariable logistic regression model

**Fig. 3:**
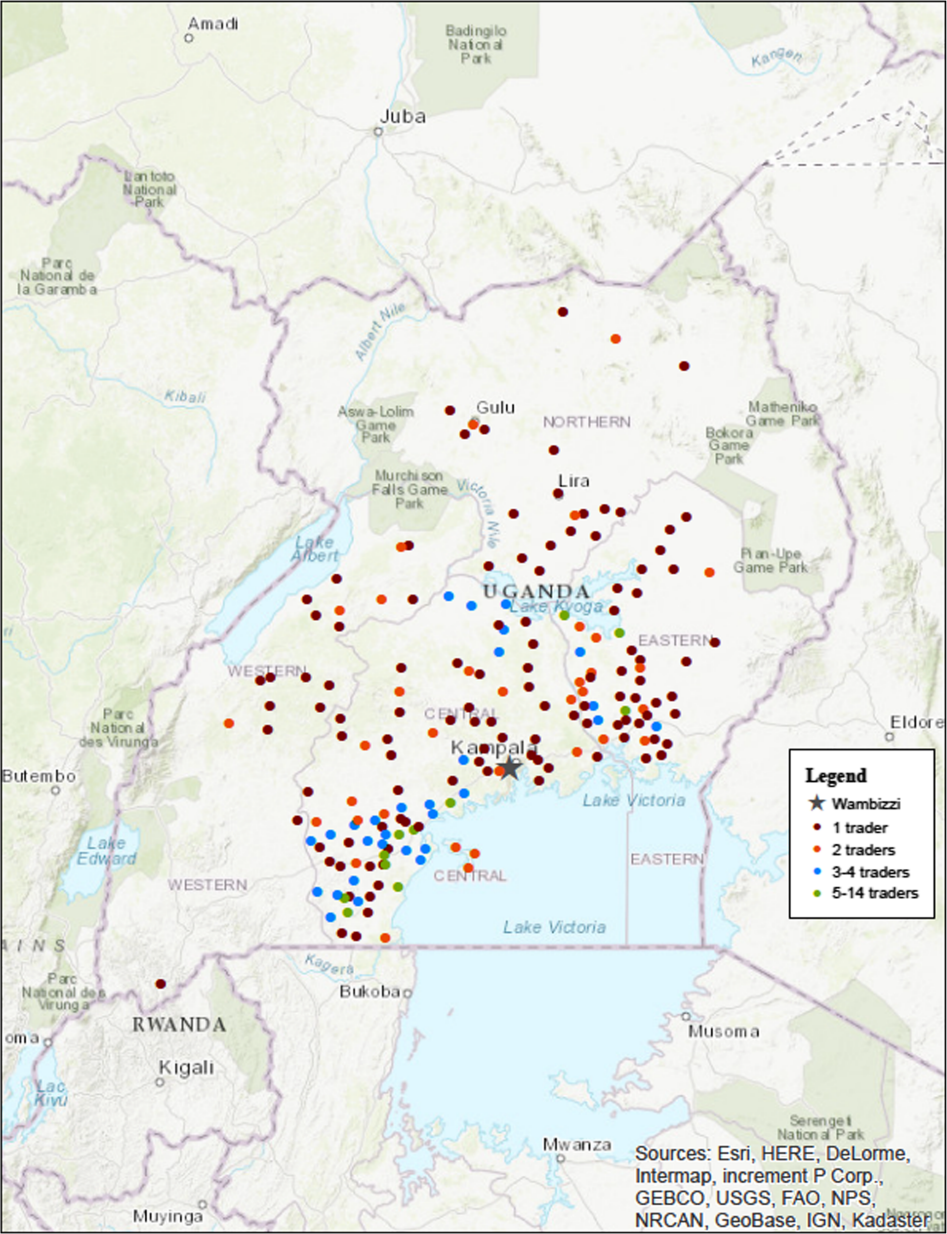
Source locations of live pigs on the day of interview and preceding 12 months, as reported by 63 pig traders interviewed at Wambizzi Cooperative Society slaughterhouse, Kampala, Uganda, 2015–2016

Figure 3 shows the source locations of pigs purchased on the day of interview and preceding 12 months. Pig traders reported buying live pigs in one to eight districts (median 3) across one to three regions (median 1) in Uganda. Thirty-six percent of traders purchased live pigs only in the central region (*n* = 23) and 27% of traders purchased live pigs only in the eastern region (*n* = 17).

Farm gate prices by operating region as well as the number of districts/regions that a pig trader operates in are outlined in Table 4. Pig traders operating in one region paid significantly higher prices per kilogram at farm gate than traders operating in two to three regions (*P* = 0.001).

**Table 4:** Farm gates prices paid by 63 pig traders interviewed at Wambizzi Cooperative Society slaughterhouse, Kampala, Uganda, 2015–2016. Non-parametric tests were used to test for significant differences by operating region, number of districts, and number of regions that a trader operates in

	High price paid per kilogram					Low price paid per kilogram				
	Number (*n*)	Lower quartile (UGX)	Median (UGX)	Upper quartile (UGX)	*P* value	Number (*n*)	Lower quartile (UGX)	Median (UGX)	Upper quartile (UGX)	*P* value
Operating region of pig trader^a^					0.05^a^					0.10^a^
Central	23	6267.6	6000	6819.4	^b^	22	5102.4	5000	5788.5	^c^
Eastern	17	6369.4	6500	6901.2	^b^	17	5264.9	5500	5793.9	^c^
All other regions	23	6020.2	6000	6571.2	^b^	23	5031.9	5000	5333.3	^c^
Missing	0					1				
Number of districts pig trader operates in^d^										
1–3 districts (< median)	31	6464.5	6500	6929.1	0.002^d^	30	5319.9	5500	5833.5	0.004^d^
4–8 districts (> median)	30	6055.4	6000	6444.6		30	5018.9	5000	5281.1	
Missing	2					3				
Number of regions pig trader operates in^d^										
1 region (< median)	42	6448.5	6500	6851.5	0.001^d^	41	5250.8	5000	5666.2	0.12^d^
2–3 regions (> median)	19	5943.6	6000	6245.9		19	5017.5	5000	5298.3	
Missing	2					3				

^a^Kruskal-Wallis test

^b^Between central and eastern region (*P* = 0.55), between eastern and all other regions (0.01), between central and all other regions (0.10).

^c^Post hoc pairwise testing was not done as operating region was *P* > 0.05.

^d^Mann-Whitney *U* test (missing values were excluded from the comparison)

The number of live pigs purchased per week by operating region and number of districts/regions that a trader operates in is shown in Table 5. During months when demand for pork was low, the region(s) a pig trader operated in to purchase live pigs was significantly associated with the number of pigs purchased (*P* = 0.014). Pig traders operating in only central region purchased a significantly higher number of pigs during low demand months than traders operating in only eastern region (*P* < 0.001). Traders operating in only the eastern region purchased a significantly lower number of pigs during low demand months than traders operating in all other regions (*P* = 0.002).

**Table 5:** Number of live pigs purchased per week by 63 pig traders interviewed at Wambizzi Cooperative Society slaughterhouse, Kampala, Uganda, 2015–2016. Non-parametric tests were used to test for significant differences by operating region, number of districts, and number of regions that a trader operates in

	Number of live pigs bought during high demand weeks					Number of live pigs bought during low demand weeks				
	Number (*n*)	Lower quartile	Median	Upper quartile	*P* value	Number (*n*)	Lower quartile	Median	Upper quartile	*P* value
Operating region of pig trader^a^					0.19^a^					0.01^a^
Central	23	71.6	80	133.2	^b^	23	30.88	30	59.55	^c^
Eastern	17	51.0	60	75.5	^b^	17	15.63	20	30.01	^c^
All other regions	21	69.1	90	128.1	^b^	21	25.33	30	56.77	^c^
Missing	2					2				
Number of districts pig trader operates in^d^										
1–3 districts (< median)	31	57.6	60	90.2	0.07^d^	31	18.91	20	30.63	< 0.001^d^
4–8 districts (> median)	30	80.6	85	8.9		30	37.13	37.5	64.34	
Missing	2					2				
Number of regions pig trader operates in^d^										
1 region (< median)	42	70.1	70	106.6	0.76^d^	42	25.26	25	43.21	0.15^d^
2–3 regions (> median)	19	62.1	80	126.4		19	28.39	35	61.30	
Missing	2					2				

^a^Kruskal-Wallis test

^b^Post hoc pairwise testing was not done as operating region was *P* > 0.05.

^c^Between central and eastern region (< 0.001), between eastern and all other regions (0.002), between central and all other regions (0.783).

^d^Mann-Whitney *U* test (missing values were excluded from the comparison)

### Knowledge and practices towards pig disease and health reporting

All the pig traders reported recognizing clinical signs indicating a pig was sick. When asked to list these signs, the most commonly stated signs were dropping of ears (46%; 29/63), reddening of ears (44.4%; 28/63), straightening of the tail (31.7%; 20/63), and weakness or difficulty standing (31.7%; 20/63). Traders typically did not report pigs considered to be sick to anyone (92.1%; 58/63). If there was reporting, the trader informed a meat inspector on site at Wambizzi (80%; 4/5) or a veterinary officer (20%; 1/5). If sick pigs were observed while under the traders’ care, 77.8% of the traders did nothing to care for the sick pigs (49/63). If action was taken, the sick pig was slaughtered at Wambizzi and the meat sold (14.3%; 9/63).

## Discussion

This is the first study to describe Ugandan pig trader characteristics and business practices around live pig buying, transportation, and health management. The prices paid to farmers for their pigs were associated with the number of regions and districts a pig trader operates in. In addition, pig traders reported paying higher prices during holiday periods and harvest season (crops/coffee). Traders preferred buying live pigs from male farmers because they considered them the final decision makers and owned the pigs being sold. Finally, we found that all pig traders checked for clinical signs in pigs that indicated the animal was sick.

Pig traders who operate in only one region paid, on average, higher prices per kilogram to farmers for their pigs. Considering that such traders are likely to travel shorter distances to source their pigs, compared to those traders that work in more regions, this may suggest that the distance travel to source pigs has an impact on the price paid for the pigs, with traders traveling less offering higher prices to farmers. However, another possibility may be that traders who operate in multiple regions are large-scale traders, buying pigs in bulk, and therefore paying lower prices. Nevertheless, given the importance of pigs as an asset within smallholder farming households, it is advantageous when pig farmers can secure more income from the sale of their pigs.

Given that almost half the traders in this study operated in four or more districts in Uganda, pigs are traveling large distances from farm to slaughterhouse. Reducing the distance pigs’ travel for processing is both an animal welfare and a disease mitigating practice, especially for limiting the dissemination of ASF (Tejler 2012). Thus, there is a need for locally regulated slaughter facilities and/or improved transport infrastructure throughout the country to reduce the distance traveled from farm to slaughterhouse. Centralized slaughter facilities would also help address the lack of consistent market access, a commonly cited constraint among pig farmers (Ouma et al. n.d.; Wabacha et al. 2004; Kagira et al. 2010; Muhanguzi et al. 2012a). Moreover, traders in this study reported that the primary reason for maintaining a fixed business operation was proximity to pork customers. Local slaughter facilities would provide a centralized location for traders to access customers.

This study also found that holiday periods, harvest season, and drought were the most commonly cited reasons trader paid high prices at farm gate. Other studies have noted that smallholder farmers keep pigs as a source of cash in times of need (Dione et al. 2014; Deka et al. 2007; Gichohi et al. 1988). When pig traders need to source live pigs around harvest season, they pay more for these pigs because farmers have recently sold their crops and, therefore, do not need the additional cash generated from the sale of a pig. The situation is a little different around the holidays. An increase in pig sales and pork consumption during festive seasons is well documented (Roesel et al. 2016; Dione et al. 2014; Adams et al. 2012; Kambashi et al. 2014). It is possible that traders have a harder time sourcing the number of pigs they need at the holiday period or that farmers know of the increased holiday demand and raise their prices. The higher prices paid for pigs around holidays are inducements for farmers to time their pig rearing activities to take advantage of the economic benefit of having pigs ready for sale according to holiday periods.

Free ranging, tethering, and feeding of crop residues and grasses to pigs are common practices among smallholder pig farmers in East Africa (Dione et al. 2015; Chenais et al. 2015; Tejler 2012; Kagira et al. 2010; Dione et al. 2014; Muhanguzi et al. 2012b; Nantima et al. 2016). In all these production systems, drought would reduce the amount and quality of feed available for pigs and, therefore, would reduce the number of pigs suitable for sale. It is also possible that farmers intentionally chose not to rear grower and fattener pigs during known seasons of drought. It will be important to address live pig supply issues, whether at holiday periods, drought, or from other causes, to ensure a consistent pork supply so that consumers are able to access the quality and quantity of pork they demand.

At the farm level, this research found that pig traders have a strong preference to buy live pigs from male pig farmers. A study in Kenya found that the decision to keep pigs was made by men (Simiyu and Foeken 2013). Because of the large financial investment, technical knowledge required to raise pigs and role as sole decision makers in their home, men maintained control of pigs and leveraged this control for any financial decisions made over the animals (Simiyu and Foeken 2013). These subtle cultural values around livestock ownership and household financial decision making come into play in accessing markets for livestock and livestock products. Women tend to face more challenges than men in accessing and benefiting from markets, especially more formal markets (Kristjanson et al. 2010). Furthermore, given women’s traditional responsibility for household food security, their level of control over decisions about whether to sell or consume the family’s animal products, as well as over how to use any income obtained from the sale of animal foods, could greatly determine the nutritional well-being of household members (Kristjanson et al. 2010). While there is clearly a need for support of women at the household level in accessing markets for their livestock, this research shows that there is also a need to work with pig traders to enable female pig farmers to access consistent markets for their pigs.

In this study, all the traders interviewed observed clinical signs they described as indicating a pig was sick. The frequently observed clinical signs such as reddening of the ears, dropping of ears, and weakness or affected movements are consistent with clinical signs of ASF (Chenais et al. 2015; Tejler 2012; Dione et al. 2014). Despite recognition of these signs as indicators of sickness, traders failed to report these suspected cases to the proper authorities. Similar findings have been reported in previous research in Uganda (Dione et al. 2015; Chenais et al. 2015; Muhangi et al. 2014; Nantima et al. 2016) and are consistent with studies conducted in Indonesia (Leslie et al. 2016). Given that traders play an essential role in transporting pigs, there is a need to develop policies and strategies to integrate pig traders into disease reporting and disease mitigating strategies without fear of recrimination or detriment to their business. In addition, given the pressures pig traders are under to meet quality standards of pork customers, pig traders would benefit from training on disease mitigating strategies including safe and hygienic slaughter practices, perhaps through an industry association or group. This would also address the gap between traders admitting that they are responsible for conducting their business in support of disease prevention but do not perceive themselves as key actors in the control of disease (Dione et al. 2016).

There are several limitations to this study. First, responses to the questionnaire are subject to recall bias. This is especially true of answers around the number and location of pigs purchased over the 12 months prior to the interview. However, the number of pigs for which traders provided a source location (*n* = 7185) was considerably less than the number of pigs processed at the slaughterhouse (*n* = 19,021 for July 2011–June 2012) (Roesel et al. 2016). It appears that when the pig traders were unsure of actual numbers, they underreported, or only reported the location they sourced the pigs without any accompanying number of pigs purchased. Self-reported locations are likely to be reliable as the traders used community-based scouts to identify pigs for sale.

We purposely interviewed pig traders during periods when demand for pork was historically high and theorized that this would mean that more pig traders would be bringing pigs in for processing. However, it is possible that there are pig traders that only operate sporadically and would have been missed in this study. We worked with the pig traders at Wambizzi to identify other pig traders to interview. We also cataloged the brands on each pig being processed, as each trader has a unique symbol to identify their pigs once they have been processed. Every effort was made to identify all potential research participants. Previous research, undertaken with the management at Wambizzi, stated that there were 20 pig traders regularly operating from the premises (Roesel et al. 2016). Given this, our study team managed to identify three times the number of pig traders.

Further analysis beyond descriptive analyses was hindered by the low number of responses for certain variables. For example, we were unable to analyze why pig traders prefer buying live pigs from men rather than women. Given the priority of gender empowerment in Uganda and the significance of livestock in alleviating poverty for women and children, it is important to identify ways to support women in accessing markets for their pigs. More detailed interviews and focus groups may shed additional light on trader buying decisions.

This study has provided baseline information on pig trader practices in Uganda. The prices paid at farm gate for pigs are affected by the number of regions and districts a trader operates in to procure pigs. Given the animal welfare and disease transmission implications of pigs traveling over multiple districts and regions from farm to slaughterhouse, consideration should be given to establishment of local pork slaughterhouses and markets and improvements to transport infrastructure. Furthermore, pig traders prefer buying live pigs from male farmers. For women to overcome the challenges of accessing formal livestock markets, there is a need for additional research to identify how women can access pork markets in Uganda, particularly if pig traders are involved. Finally, this research shows that pig traders are observing sick pigs but fail to report these sick pigs. Historically, disease control interventions have been focused on farm level biosecurity. Given their role as a link between farmers and consumers, traders would benefit from targeted inclusion in disease control and prevention strategies.

## Electronic supplementary material

ESM 1 (DOCX 73 kb)
