# Supplementary material for: Analysis of pig trading networks and practices in Uganda
Source: Trop Anim Health Prod. 2018 Aug 2;51(1):137–47. doi: 10.1007/s11250-018-1668-6 (PMC6347582; doi:10.1007/s11250-018-1668-6)
Supplement: Supplementary file 1 — (DOCX 73 kb) [file 11250_2018_1668_MOESM1_ESM.docx]

**Section A: Background Information**

| 1. Date of survey (DD/MM/YYYY) |  |
| --- | --- |
| 1. Enumerator Name: |  |
| 1. Sex of the respondent: | Male / Female |
| 1. Age of the respondent (years) |  |
| 1. What is the highest level of education completed | School not attended Diploma/certificate  Primary not completed Undergraduate university degree  Primary school Postgraduate university degree  Secondary school Other (specify):________________________________________ |
| 1. How long have you been a pig trader | ________ years / months / weeks (please circle unit) |
| 1. From whom do you commonly prefer buying pigs | Men / Women |
| 1. Give reasons for our preference above (Q7) | 1._____________________________________________________________________________________________  2._____________________________________________________________________________________________  3. ____________________________________________________________________________________________ |
| 1. Do you belong to a group or cooperative? | Yes / No  If yes, name of group or cooperative:______________________________________________________________ |

**Section B: Trade details**

| 1. What is your position in the business | Owner Relative of owner  Employee Other (specify):__________________________ | | | |
| --- | --- | --- | --- | --- |
| 1. Is your business location fixed or mobile | Fixed / mobile | | | |
| 1. If fixed, what factors contributed to your choice of location | Close to purchase points no competition  Close to customers (demand areas) no choice  No competition Other (specify)________________________________ | | | |
| 1. Are there other pig traders doing the same type of business that you do in your area of operation | Yes / No | | | |
| 1. Why do you sell pigs | Primary income Family tradition  Secondary income Other (specify):______________________________________ | | | |
| 1. Where do you sell pigs | Wambizzi  Other (specify):_________________________________________ | | | |
| 1. The pigs you are selling today are from | Your farm a smallholder farm  A neighbor’s farm from a market (specify location):______________________  A commercial farm other (specify):___________________________________ | | | |
| 1. Where did the pigs come from that you brought to Wambizzi for processing today? | District | Subdistrict | Village | # of pigs |
|  |  |  |  |  |
|  |  |  |  |  |
|  |  |  |  |  |
|  |  |  |  |  |
|  |  |  |  |  |
| 1. In the past 12 months, what other locations have you bought pigs from that were processed at Wambizzi? | District | Subdistrict | Village | # of pigs |
|  |  |  |  |  |
|  |  |  |  |  |
|  |  |  |  |  |
|  |  |  |  |  |
|  |  |  |  |  |
|  |  |  |  |  |
|  |  |  |  |  |
|  |  |  |  |  |
|  |  |  |  |  |
|  |  |  |  |  |
|  |  |  |  |  |
| **Thinking back over the last 12 months, please answer questions 19-30 accordingly** | | | | |
| 1. What months have the highest demand for pigs to be processed at Wambizzi (circle all that apply) | January May September  February June October  March July November  April August December | | | |
| 1. What months have the lowest demand for pigs to be processed at Wambizzi (circle all that apply) | January May September  February June October  March July November  April August December | | | |
| 1. During the months of low demand for pigs, how many pigs do you buy per week |  | | | |
| 1. During months of high demand for pigs, how many do you buy per week |  | | | |
| 1. What is the lowest price paid per pig at farm | __________ per kg / per animal (circle unit) | | | |
| 1. What months are prices per pig the lowest (circle all that apply) | January May September  February June October  March July November  April August December | | | |
| 1. When prices are lower for pigs, this is due to | Holidays Sick pigs  School fees Neighbor’s pigs are sick  Drought Other (specify):____________________________________ | | | |
| 1. What is the highest price paid per pig at farm | __________ per kg / per animal (circle unit) | | | |
| 1. What months are prices per pig highest | January May September  February June October  March July November  April August December | | | |
| 1. When prices are higher for pigs, this is due to | Holidays Sick pigs  School fees Neighbor’s pigs are sick  Drought Other (specify):____________________________________ | | | |
| 1. What is the best time of day for buying pigs | Morning Afternoon  Midday Night | | | |
| 1. Why is this the best time of day for buying pigs? | Better prices Allows time to travel to Wambizzi  More pigs available Other(specify):_________________________________ | | | |
| 1. How do you transport live pigs   (circle all that apply) | Car Boda boda  Truck Matatu  Lorry Boat | | | |
| 1. Who owns the vehicle you are using to transport pigs today | Own vehicle borrowed rented | | | |
| 1. How often do you clean up animal waste and feces from the vehicle you use to transport pigs | After each use every month  Every day never  Every week | | | |
| 1. Which of the waste disposal methods do you most commonly use for removal of animal waste and feces from your vehicle | Bury Cover waste with soil, sand or ash  Burn Compost  Clean by hand Throw away  Other (specify):_____________________________________________ | | | |
| 1. Are any products used for cleaning pig waste and feces from your vehicle? | No Jik  Water Soap (detergent, Omo)  Other (specify):______________________________________________________________ | | | |
| 1. Are there symptoms you are aware of that indicate that a pig is sick | Yes / No | | | |
| 1. If yes to Q36 above, please specify from the options below. Tick only what the respondent states; multiple answers are possible | Coughing Vomiting  Weak Fever  Red eyes Depression/lethargy  Diarrhea Dropping ears  Skin flash Parasites (lice, ticks, fleas, mites)  Other (specify):_________________________________________________________ | | | |
| 1. If a pig is sick while in your care, what is usually done | Nothing slaughtered and meat is sold  Treated with medicine slaughtered and meat consumed by family | | | |
| 1. Are dead or sick pigs under your care reported to anyone | Sick pig: yes / sometimes / not sure / no  Dead pig: yes / sometimes / not sure / no | | | |
| 1. If reported, please specify who this information is reported to | Meat inspector on site Wambizzi management  Other (specify):___________________________________________________________ | | | |
